# miRNA-guided reprogramming of glucose and glutamine metabolism and its impact on cell adhesion/migration during solid tumor progression

**DOI:** 10.1007/s00018-022-04228-y

**Published:** 2022-03-29

**Authors:** Lorena Quirico, Francesca Orso, Stefania Cucinelli, Mladen Paradzik, Dora Natalini, Giorgia Centonze, Alberto Dalmasso, Sofia La Vecchia, Martina Coco, Valentina Audrito, Chiara Riganti, Paola Defilippi, Daniela Taverna

**Affiliations:** 1 Molecular Biotechnology Center (MBC), University of Torino, Via Nizza, 52, 10126 Torino, Italy; 2 Department Molecular Biotechnology and Health Sciences, University of Torino, Via Nizza, 52, 10126 Torino, Italy; 3 Department of Oncology, University of Torino, Torino, Italy; 4 Present Address: Laboratory for Mitochondrial Bioenergetics and Diabetes, Division of Molecular Medicine, Ruđer Bošković Institute, Zagreb, Croatia

**Keywords:** miRNAs, Metabolism, Cancer, Metastasis, Adhesion

## Abstract

MicroRNAs (miRNAs) are small, non-coding RNAs about 22 nucleotides in length that regulate the expression of target genes post-transcriptionally, and are highly involved in cancer progression. They are able to impact a variety of cell processes such as proliferation, apoptosis and differentiation and can consequently control tumor initiation, tumor progression and metastasis formation. miRNAs can regulate, at the same time, metabolic gene expression which, in turn, influences relevant traits of malignancy such as cell adhesion, migration and invasion. Since the interaction between metabolism and adhesion or cell movement has not, to date, been well understood, in this review, we will specifically focus on miRNA alterations that can interfere with some metabolic processes leading to the modulation of cancer cell movement. In addition, we will analyze the signaling pathways connecting metabolism and adhesion/migration, alterations that often affect cancer cell dissemination and metastasis formation.

## Introduction

MicroRNAs (miRNAs) are small non-coding RNAs that, at a post-transcriptional level silence their target mRNAs via translational repression or mRNA degradation. They are mainly located in introns or exons of protein-coding genes, as well as in intergenic regions. Most of them are transcribed in the nucleus by RNA polymerase II, generating a long primary miRNA (pri-miRNA), which is then processed by the DROSHA-DGCR8 complex to release a precursor-miRNA (pre-miRNA). Exportin 5 subsequently translocates the pre-miRNA to the cytoplasm where it is cleaved by the RNase III Dicer to form a mature miRNA. It is eventually loaded onto Argonaute family (AGO) proteins to form the effector RNA-induced silencing complex (RISC) [1]. miRNAs are master regulators of protein-coding gene expression: indeed, more than 60% of human genes have, at least, one conserved miRNA binding site [2]. Moreover, each miRNA may have hundreds of targets and a single mRNA may be regulated by several miRNAs. Therefore, alterations in their expression are often associated with diseases, including cancer in which they act as tumor suppressors or oncomiRs. The first link between miRNAs and tumors refers to the miR-15/16 cluster in Chronic Lymphocytic Leukemia [3]. Up to now, a many alterations in miRNA expression have been identified in tumors [4] and have been related to cancer hallmarks [5]. In particular, miRNAs are well-known regulators of adhesion, invasion, metastatic process [6] and recently, emerging evidence supports their role in the regulation of cancer metabolism.

**Fig. 1:**
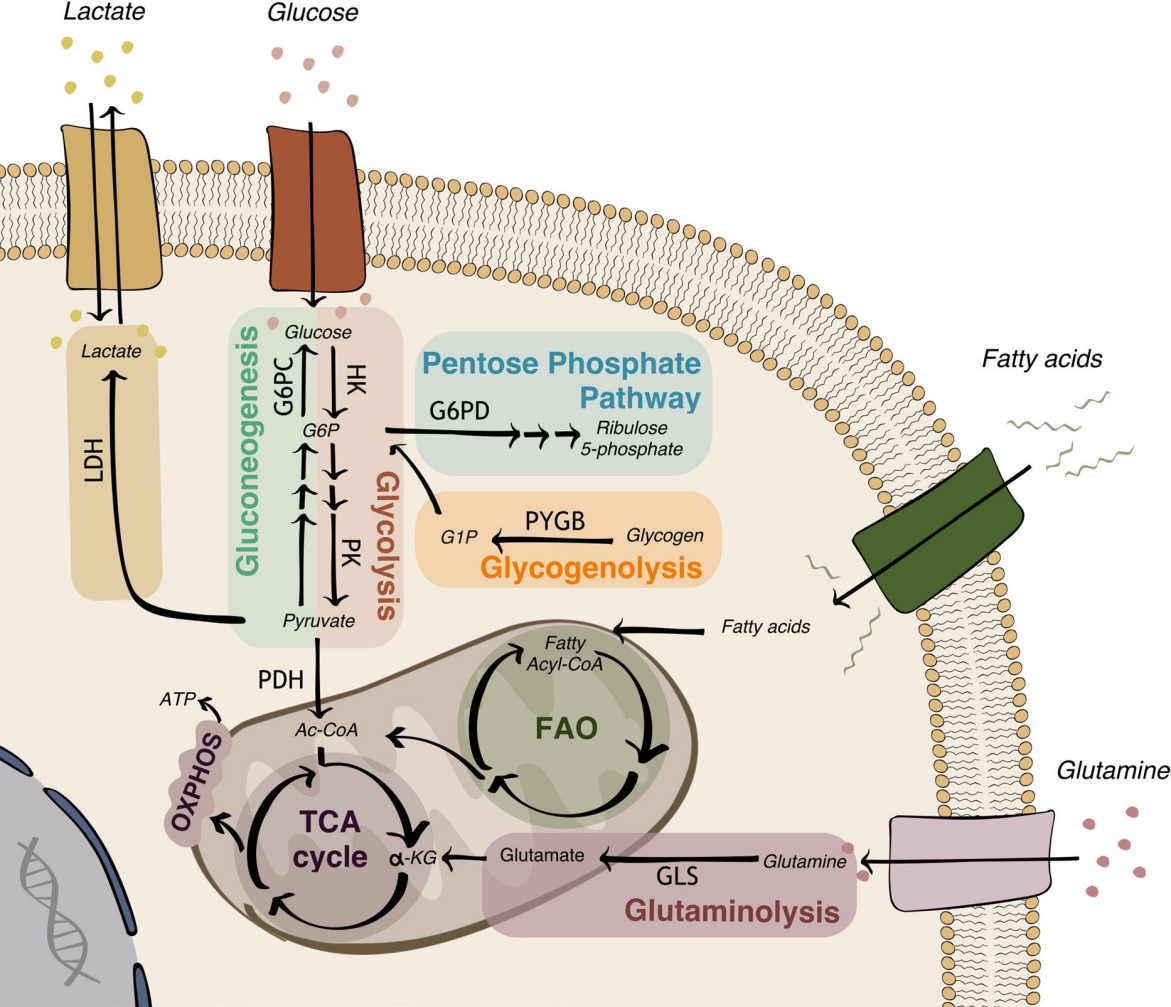
Main metabolic pathways in cancer cells. The figure represents a schematic overview of the main metabolic pathways and enzymes in cancer cells. *α-KG* alpha-ketoglutarate, *Ac-CoA* acetyl-CoA, *ATP* adenosine triphosphate, *FAO* fatty acid *β*-oxidation, *G1P* glucose-1 phosphate, *G6P* glucose-6 phosphate, *G6PC* glucose-6-phosphatase, *G6PD* glucose-6-phosphate-dehydrogenase, *GLS* glutaminase, *HK* hexokinase, *LDH* lactate dehydrogenase, *OXPHOS* oxidative phosphorylation, *PDH* pyruvate dehydrogenase, *PK* pyruvate kinase, *PYGB* glycogen phosphorylase B, *TCA cycle* tricarboxylic acid cycle

Cell metabolism is the sum of reactions supporting cell survival, proliferation, migration, resiliency to noxious stresses and the control of oxido-reductive balance. One century ago, Warburg observed that cancer cells use glycolysis as the main energetic pathway, either in the presence or absence of oxygen [7]. Consequently, the reprogramming of cell metabolism depending on the environmental conditions is considered a cancer hallmark [8]. Although the Warburg effect fuels cancer cells to metastasize [9], mitochondrial-related metabolic pathways are not necessarily defective in neoplasia. Many cancers rely on oxidative phosphorylation (OXPHOS) and aerobic mitochondrial metabolism [10], based on a tricarboxylic acid (TCA) cycle and fatty acid *β*-oxidation (FAO). Moreover, to survive in poor nutrient and hypoxic conditions, cancer cells use opportunistic nutrient acquisition strategies as, for example, the uptake of amino acids via micropinocytosis or apoptotic bodies [11]. Since histone acetylation alters the expression of oncosuppressors, epigenetic alterations are also associated with changes in metabolism. Acetyl-CoA (Ac-CoA) is the substrate for histone acetylases [12] and proteins controlling tumor progression [13]. Thus, a metabolic drift towards increased cytosolic Ac-CoA production may increase histone acetylation and confers proliferative/promigratory phenotypes in cancer cells [14]. It is important to note that tumors are characterized by metabolic plasticity: cancer cells may process the same substrate in different ways and use distinct energy sources to adapt to the tumor microenvironment (TME). In addition, metabolic heterogeneity within the same tumor mass further complicates the picture. As it has been demonstrated, metabolic rewiring in cancer cells is driven both by intrinsic and extrinsic factors, and affects not only energy production and biomolecule synthesis, but also cancer biology extensively [15]. Figure 1 highlights the main metabolic pathways in cancer cells.

In healthy tissues, homeostasis mainly depends on adhesion between cells and the extracellular matrix (ECM). These interactions are mediated by a large group of transmembrane receptors, generically defined as Cell Adhesion Molecules (CAMs), which include the calcium-dependent integrins, cadherins and selectins as well as the calcium-independent Immunoglobulin superfamily CAMs [16]. The same adhesion receptors control proliferation, tissue integrity and cell migration and are able to activate signaling cascades and mechano-signals [17]. Therefore, any abnormal change in cell adhesion can lead to malignant transformation and tumor progression [18]. In this context, miRNAs are pivotal regulators of adhesion receptor expression, which impacts tumor progression.

The aim of the present review is to discuss how miRNAs are involved in the regulation of glucose and glutamine metabolism and in the expression of adhesion molecules during cancer progression. In addition, we dissect the interplay between adhesion/migration and metabolism in malignancy and finally we discuss how miRNAs interfere with these hallmarks of cancer.

## How miRNAs tightly regulate cell metabolism

**Fig. 2:**
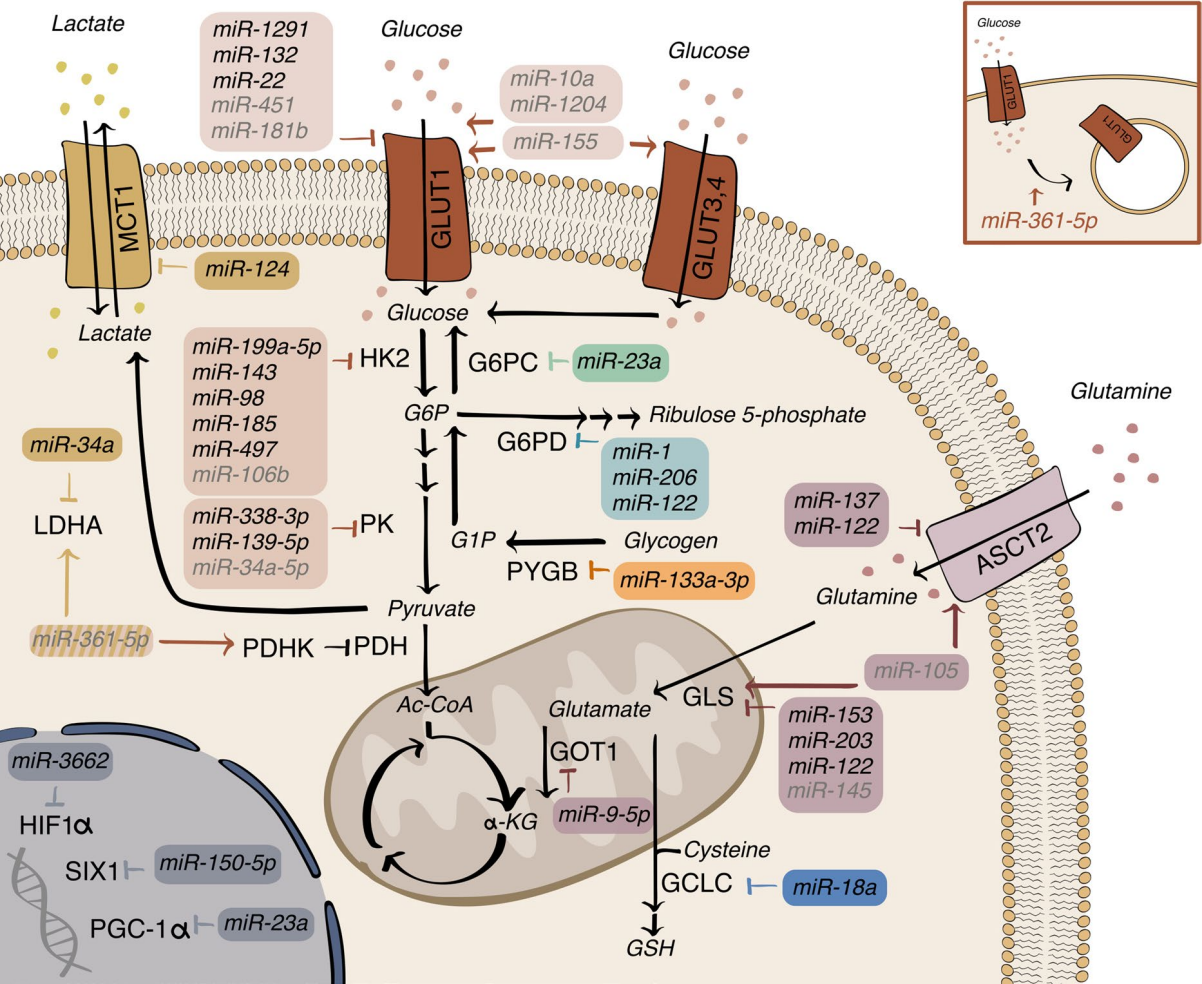
miRNAs as tight regulators of cell metabolism. The illustration shows the involvement of miRNAs in the regulation of metabolic processes in cancer cells. *α-KG* alpha-ketoglutarate, *Ac-CoA* acetyl-CoA, *ASCT2* alanine, serine, cysteine and glutamate transporter, *G1P* glucose-1 phosphate, *G6P* glucose-6-phosphate, *G6PC* glucose-6-phosphatase, *G6PD* glucose-6-phosphate-dehydrogenase, *LC* glutamate-cysteine ligase catalytic subunit, *GLS* glutaminase, *GLUT* glucose transporter, *GOT1* glutamate-oxaloacetate transaminase, *GSH* glutathione, *HIF1α* hypoxia-inducible factor 1-alpha, *HK2* hexokinase2, *LDHA* lactate dehydrogenase A, *MCT1* monocarboxylate transporter 1, *PDH* pyruvate dehydrogenase, *PDHK* pyruvate dehydrogenase kinase, *PGC-1α* peroxisome proliferator-activated receptor gamma coactivator 1-alpha, *SIX1* sine oculis homeobox 1, *PK* pyruvate kinase, *PYGB* glycogen phosphorylase B. *Black miRNAs* direct targeting, *gray miRNAs* indirect targeting. *Blocking arrows* block by a miRNA, *arrows* final activation by miRNAs

The metabolic reprogramming of cancer cells is tightly regulated at transcriptional or post-transcriptional levels, where miRNAs play important roles [19]. Notably, both tumoral or stromal miRNAs may operate here, making the metabolic crosstalk within the TME highly variegate [20]. Since glucose and glutamine metabolism strongly promotes cancer cells, a high amount of lactate and their derivatives are released in the extracellular milieu, affecting the TME composition and favoring angiogenesis and tumor progression [21]. A research that used over 6000 tumors revealed miR-34a-5p, miR-106b-5p, miR-146a-5p and miR-155-5p as the universal controllers of cancer metabolism, thus defining them as metabomiRs [22]. However, other miRNAs have been found involved in the control of cancer metabolic pathways. Here, we focus on the link between glucose-related pathways or glutaminolysis and miRNAs in cancer cells (Fig. 2 and Table 1).

**Table 1:** miRNAs involved in cancer cell metabolic pathways

miRNA	Direct target	Pathway	Cancer type	Reference
miR-1291	GLUT1	Glucose uptake	Renal cell carcinoma	[25]
miR-451	CAB39	Glucose uptake Glycolysis	Glioma	[26]
miR-181b	SP1	Glucose uptake	Glioma	[27]
miR-10a		Glucose uptake	Oral squamous cell carcinoma	[28]
miR-1204		Glucose uptake	Ovarian squamous cell carcinoma	[29]
miR-132	GLUT1	Glucose uptake	Prostate cancer	[30]
miR-22	GLUT1	Glucose uptake	Breast cancer	[31]
miR-155	PIK3R1, FOXO3a	Glucose uptake Glycolysis	Breast cancer	[32]
miR-361-5p	FGFR1	Glucose uptake Glycolysis	Breast cancer	[33]
miR-199a-5p	HK2	Glycolysis	Liver cancer	[34]
miR-143	HK2	Glycolysis	Oral squamous cell carcinoma	[35]
miR-98	HK2	Glycolysis	Colorectal cancer	[36]
miR-185	HK2	Glycolysis	Osteosarcoma	[37]
miR-497	HK2	Glycolysis	Osteosarcoma	[38]
miR-338-3p	PKM2	Glycolysis	Ovarian cancer	[39]
miR-139-5p	PKM2	Glycolysis	Gallbladder carcinoma	[40]
miR-34a	LDHA	Glycolysis	Breast cancer	[41]
miR-34a-5p		Glycolysis	Breast cancer	[42]
miR-124	MCT1	Glycolysis	Pancreatic ductal adenocarcinoma	[43]
miR-150-5p	SIX1	Glycolysis	Melanoma	[44]
miR-3662	HIF-1*α*	Glycolysis	Hepatocellular carcinoma	[45]
miR-106b	PLK3	Glycolysis	Prostate cancer	[46]
miR-1	G6PD, PGD, TKT	Pentose phosphate pathway	Lung cancer, cervical cancer, pituitary cancer, hepatocellular carcinoma	[48, 50–51]
miR-206	G6PD, PGD, TKT	Pentose phosphate pathway	Lung cancer Cervical cancer	[48–49]
miR-122	G6PD	Pentose phosphate pathway	Hepatocellular carcinoma	[52, 66]
	ACT2, GLS	Glutaminolysis		
miR-23a	PGC-1*α* G6PC	Gluconeogenesis	Hepatocellular carcinoma	[53]
miR-133a-3p	PYGB	Glycogenolysis	Ovarian cancer	[54]
miR-137	ASCT2	Glutaminolysis	Glioblastoma, colorectal cancer, pancreatic ductal adenocarcinoma, prostate cancer	[59]
miR-153	GLS	Glutaminolysis	Glioblastoma	[60]
miR-203	GLS	Glutaminolysis	Melanoma	[61]
miR-145	c-Myc	Glutaminolysis	Ovarian cancer	[62]
miR-18a	GCLC	Glutaminolysis	Liver cancer	[63]
miR-9-5p	GOT1	Glutaminolysis	Pancreatic cancer	[65]
miR-105	MXI1	Glutaminolysis, glycolysis	Breast cancer	[67]

### Glucose uptake and glucose-related pathway

Cancer cells show an enhanced glucose uptake and an alteration of glucose-related pathways including glycolysis, pentose phosphate pathway (PPP), gluconeogenesis and glycogenolysis compared to their normal counterparts. These aspects will be reviewed in the next paragraphs.

#### Glucose uptake

The glucose transporters (GLUTs) move the glucose through the plasma membrane by means of facilitated diffusion. GLUT1 is the predominant carrier and its overexpression is associated with malignancy and poor prognosis in cancer [23, 24], where it can be controlled by miRNAs. For instance, in renal cell carcinomas (RCC), GLUT1 is downregulated by miR-1291 [25]. Since miR-1291 levels are lower in tumors compared to those in surrounding normal tissues, this phenotype confers a metabolic advantage to RCC cells. In gliomas, GLUT1 is inhibited by miR-451, which targets the calcium-binding protein 39 (CAB39), thereby arresting glucose uptake and metabolism, as well as lactate production. In addition, glioma xenografts pretreated with miR-451 show a reduced GLUT1 expression compared to controls [26]. In the same tumor type, GLUT1 is also an indirect target of miR-181b. Histological analyses on miR-181b overexpressing xenografts revealed a decrease of GLUT1 expression and *viceversa* for miR-181b depleted tumors [27]. In oral squamous cell carcinomas (OSCC), GLUT1 is up-regulated by the oncogenic miR-10a which promotes glucose uptake and glucose metabolism leading to increased cell proliferation, indicating that glucose is required to sustain cancer proliferation and aggressiveness [28]. Similarly, miR-1204 levels and GLUT1 are positively correlated in biopsies of ovarian squamous cell carcinomas and not in non-transformed ovarian tissue, suggesting a specific pathway for ovarian cancer [29]. Several oncosuppressor miRNAs downregulate GLUT1 as, for example miR-132 in prostate cancer (PC), where GLUT1 is also regulated by cucurbitacin D (Cuc D), an anti-cancer plant steroid [30], thus indicating that metabolic effects may be manipulated not only with miRNA mimics or anti-miRNAs/sponges, but also with pharmacological or natural products. In addition, an inverse correlation between miR-22 and GLUT1 expression was found in breast cancer samples. Low miR-22 and high GLUT1 levels are significantly associated with a shorter disease-free survival or overall survival, thus impacting prognosis [31]. Although GLUT1 is the predominant isoform in tumors, other GLUT isoforms can also be modulated by miRNAs. In fact, Kim and colleagues showed that miR-155 knock-out leads to reduced GLUT1, GLUT3 and GLUT4 expression (32). Besides modulating GLUT1 levels, miRNAs may also relocate it. For instance, miR-361-5p regulates the translocation of GLUT1 from the plasma membrane to the cytoplasm, thus reducing anaerobic glycolysis, proliferation and invasion. In contrast, fibroblast growth factor receptor 1 (FGFR1), a miR-361-5p target, reverses GLUT1 subcellular translocation [33]. Overall, the effects of miRNAs on glucose uptake are particularly variegate.

#### Glycolysis

Glycolysis is one of the main energetic pathways in tumors, which produces 2 adenosine triphosphate (ATP) molecules when glucose is metabolized into lactate, or, a higher number of ATP units when it generates pyruvate. miRNAs control some of the main glycolytic enzymes, such as those mentioned in the following paragraphs.

The hexokinase (HK), a key glycolytic enzyme and the branching point of PPP, converts glucose into glucose-6-phosphate (G6P), so it is not surprising that HK expression is controlled by miRNAs. Indeed, in liver cancer, miR-199a-5p directly targets HK2, decreasing glucose consumption, lactate production, G6P and ATP levels. In patients with liver tumors, HK2 is upregulated and miR-199a-5p is deregulated [34]. In OSCC, miR-143 directly targets HK2, thus suppressing glycolysis and decreasing lactate dehydrogenase A (LDHA) levels and activity. Moreover, a negative correlation between miR-143 [35] or miR-98 [36] and HK2 has been detected in oral tumor tissues and in colorectal cancer (CRC), respectively. Likewise, HK2 levels negatively correlate with miR-185 [37] and miR-497 [38] in osteosarcoma samples, suggesting a multiple miRNA control within the same tumor type.

Pyruvate kinase (PK) catalyzes the transfer of a phosphate group from phosphoenolpyruvate (PEP) to ADP with the consequent formation of pyruvate and ATP. The isoform M2 (PKM2) is a direct target of miR-338-3p in ovarian cancer [39] and miR-139-5p in gallbladder carcinoma [40].

LDHA is the last glycolytic enzyme of the glycolytic pathway and is closely controlled by miRNAs. Since LDHA is a miR-34a direct target, it is negatively correlated with such miRNA in breast cancer. In addition, LDHA-induced glycolysis can be inhibited by miR-34a [41]. In the same tumor type, LDHA, is downregulated by miR-34a-5p, together with PKM2. In human breast cancer samples or TCGA datasets, low miR-34a-5p expression correlates with high LIN28B levels and, together with high MYC levels, it predicts poor survival. Interestingly, the LIN28B/MYC/miR-34a-5p pathway can be therapeutically exploited: in fact, when using LIN28B inhibitors, miR-34a-5p increases and PKM2 decreases while tumor growth and lung metastasis are suppressed [42]. In conclusion, miR-361-5p inhibits glycolysis in breast cancer cells and FGFR1, a miR-361-5p target, reverts the anti-glycolytic function of those small RNAs and represses OXPHOS by upregulating LDHA and pyruvate dehydrogenase kinase-1 (PDHK1) function. An inverse expression between miR-361-5p and FGFR1 is present in clinical samples [33]. The modulation of LDHA by miRNAs may also be coordinated by the monocarboxylate transporter 1 (MCT1), which transports lactate out of cancer cells. Indeed, miR-124 inhibits glycolysis and lactate export by targeting MCT1 in pancreatic ductal adenocarcinoma (PDAC). As a consequence, intracellular acidification slows down the glycolytic flux and LDHA activity, thus reducing tumor growth and invasion [43].

Lastly, miRNAs may also act on transcription factors (TFs) involved in aerobic glycolysis, such as sine oculis homeobox 1 (SIX1), targeted by miR-150-5p. As a matter of fact, as a consequence of SIX1 down-modulation, glycolysis is reduced due to a decrease in glucose uptake, lactate and ATP production, extracellular acidification rate (ECAR) and an increase in oxygen consumption rate (OCR) [44]. Similar results are observed for miR-3662 which suppresses glycolysis by directly targeting Hypoxia-inducible factor 1-alpha (HIF-1*α*) in hepatocellular carcinoma (HCC) [45]. Another TF involved in glycolysis and in the promotion of invasive behavior is STAT3 that in PC cells is indirectly regulated by miR-106b, suppressing HK2 transcription [46].

#### Pentose phosphate pathway (PPP)

Glycolysis can be directed to the PPP that supplies cells with nicotinamide adenine dinucleotide phosphate (NADPH), maintaining a reserve of reduced glutathione (GSH) and ribose-5-phosphate, a precursor for nucleotide synthesis [47]. Controlling both glycolysis and the flux toward the PPP is of paramount importance for the survival and/or malignancy of cancer cells. In particular, the diversion of G6P toward PPP increases tumor aggressiveness by proving ribose 5-phosphate and reductive equivalents as NADPH that are exploited in lipid biosynthesis as well as in the protection from oxidative stress. This linkage with the protection from oxidative damage is demonstrated by reduced levels of the ROS-sensitive nuclear factor erythroid 2-related factor 2 (Nrf2) in cells with high levels of glucose-6-phosphate-dehydrogenase (G6PD), the rate-limiting enzyme of the PPP pathway. The increase of G6P and the consequent decrease in Nrf2 activity is mediated by miR-1 and miR-206 in lung cancers [48]. In tumors, G6PD levels increase with tumor grade and the enzyme is targeted by miR-206 [49] and miR-1 [50] in cervical cancers associated with papillomavirus infections, whereas it is downregulated by miR-1 in pituitary tumors [51]. Moreover, in HCC samples, G6PD levels negatively correlate with miR-1 and the liver-specific miR-122, while loss of expression of these two miRNAs promotes tumor growth [52].

#### Gluconeogenesis and glycogenolysis

Gluconeogenesis is a mechanism used to maintain proper blood glucose levels, thus generating glucose from non-carbohydrate carbon molecules. It mainly occurs in the liver, and it may, therefore, be altered during liver tumorigenesis. To study gluconeogenesis in HCC, Wang et al. used a choline-deficient diet in mice and found a dramatic inhibition of the peroxisome proliferator-activated receptor gamma coactivator 1-alpha (PGC-1*α*) and glucose-6-phosphatase (G6PC) at the expense of miR-23a direct targeting. Relevantly, a negative expression correlation of PGC-1*α* and G6PC with miR-23a was also found in human specimens [53]. Alternatively, the excess of glucose may be stored as glycogen and its metabolism can be critical for cancer development. Indeed, glycogen phosphorylase B (PYGB), the rate-limiting enzyme in glycogenolysis, is involved in various tumors, including ovarian cancer where its levels are upregulated and correlate with poor prognosis. Mechanistically, PYGB is targeted by miR-133a-3p, and an indirect correlation between miRNA and enzyme is present in ovarian cancer samples [54]. Overall, as presented, miRNAs are able to affect glucose-related pathways at different levels.

### Glutaminolysis

Glutamine is the preferred amino acid of cancer cells, which fuels the TCA cycle via its oxidative metabolism [55]. It represents a key source of carbon and nitrogen for the de novo biosynthesis of nucleotides, non-essential amino acids, lipids [56, 57] and anaplerotic metabolites for the TCA cycle, lipids, nucleotides and precursors of GSH. Indeed, proliferating cancer cells display a high glutamine demand [58]. Multiple tumor cells and TME-related molecules, including miRNAs, control glutamine uptake and metabolism. Glutamine enters the cells mainly through the alanine, serine, cysteine, and glutamate transporter (ASCT2), targeted by the oncosuppressor miR-137, which inversely correlates with ASCT2 in glioblastoma, CRC, PDAC and PC. The epigenetic downregulation of miR-137 results instead in an increased glutamine uptake, enabling cancer cells to survive in an adverse environment thanks to the favorable supply of glutamine [59]. Upon entry, glutamine is converted to glutamate by glutaminase (GLS), an enzyme directly targeted by miR-153, and miR-203, frequently downregulated in glioblastoma and melanoma, respectively [60, 61]. miR-145 has recently been shown to inhibit glutaminolysis in ovarian cancer cells by down-regulating c-Myc, an event which reduces GLS transcription [62]. The decreased proliferation caused by low c-Myc levels and a reduced use of glutamine may explain why miR-145 overexpressing ovarian cancers have a low proliferation rate and aggressiveness. In addition, c-Myc may orchestrate the rewiring of glutamine metabolism *via* miR-18a upregulation. C-Myc-regulated miR-18a downregulates the glutamate-cysteine ligase catalytic subunit (GCLC), the rate-limiting enzyme of glutathione synthesis in liver cancer, reducing the availability of GSH [63]. Since active c-Myc potentially increases the availability of glutamine, conferring a selective advantage of cancer cells over non-transformed tissues, c-Myc/miR-18a-overexpressing cells are more susceptible to oxidative damages because of their inability to exploit glutamine to synthesize GSH.

To fuel the TCA cycle, glutamate is metabolized to alpha-ketoglutarate (*α*-KG) by glutamate dehydrogenase (GDH). Alternatively, it is converted to *α*-KG by transaminases, such as the glutamate-oxaloacetate transaminase (GOT1). PDAC strongly relies on GOT1 activity for sustained cell proliferation [64]. miR-9-5p acts as a tumor suppressor through GOT1 direct targeting, thus impairing PC cell proliferation and invasion and affecting the glutamine-dependent NADPH production and redox homeostasis [65]. As for glucose metabolism, a single miRNA may have multiple targets in glutamine metabolism. For instance, depletion of miR-122 in HCC shows the upregulation of ASCT2 and GLS. The ensemble of miR-122, ASCT2 and GLS may be considered a prognostic signature: so, miR-122 levels inversely correlate with ASCT2/GLS in patients, whereas high expression of ASCT2 and GLS correlates with poorer prognosis [66].

Ultimately, glutamine metabolism may also be influenced by extracellular vesicles (EVs) that play an essential role in the metabolic crosstalk between tumor cells and Cancer Associated Fibroblasts (CAFs). For instance, the triple negative breast cancer cells MDA-MB-231 release miR-105-rich EVs that force CAFs to up-regulate c-Myc, thus reprogramming CAFs metabolism to increase glutaminolysis and glycolysis. The final products of these pathways, glutamate and pyruvate or acetate, are released by CAFs and can be exploited opportunistically by cancer cells [67].

Interestingly, by inhibiting both glutamine and glucose metabolism, a synergistic effect is shown in osteosarcoma [68]. Similarly, the modulation of miRNAs that act on glucose and glutamine metabolism may represent a powerful new therapeutic approach in cancer treatment.

## miRNAs as relevant regulators of adhesion/migration/invasion processes

Integrins are a large family of adhesion receptors and the main components of focal adhesions (FAs), large molecular complexes that transmit a signal bidirectionally, from the outside to the inside of the cells and *viceversa* [69]. Importantly, FAs are highly dynamic structures that undergo continuous reorganization in response to TME stimuli including ECM alterations, growth factors and nutrient availability [70]. Changes in migration are mainly driven by the cytoskeleton, crucial for FA turnover, and composed of microtubules, actin and intermediate filaments [71]. Integrins and their associated complexes form the adhesome, consisting of ~ 200 proteins [72], whose deregulation is tightly associated with several diseases [73]. Interestingly, the adhesome components may be targeted by miRNAs, thus resulting in alteration of adhesion or motility.

### Adhesome formation

**Fig. 3:**
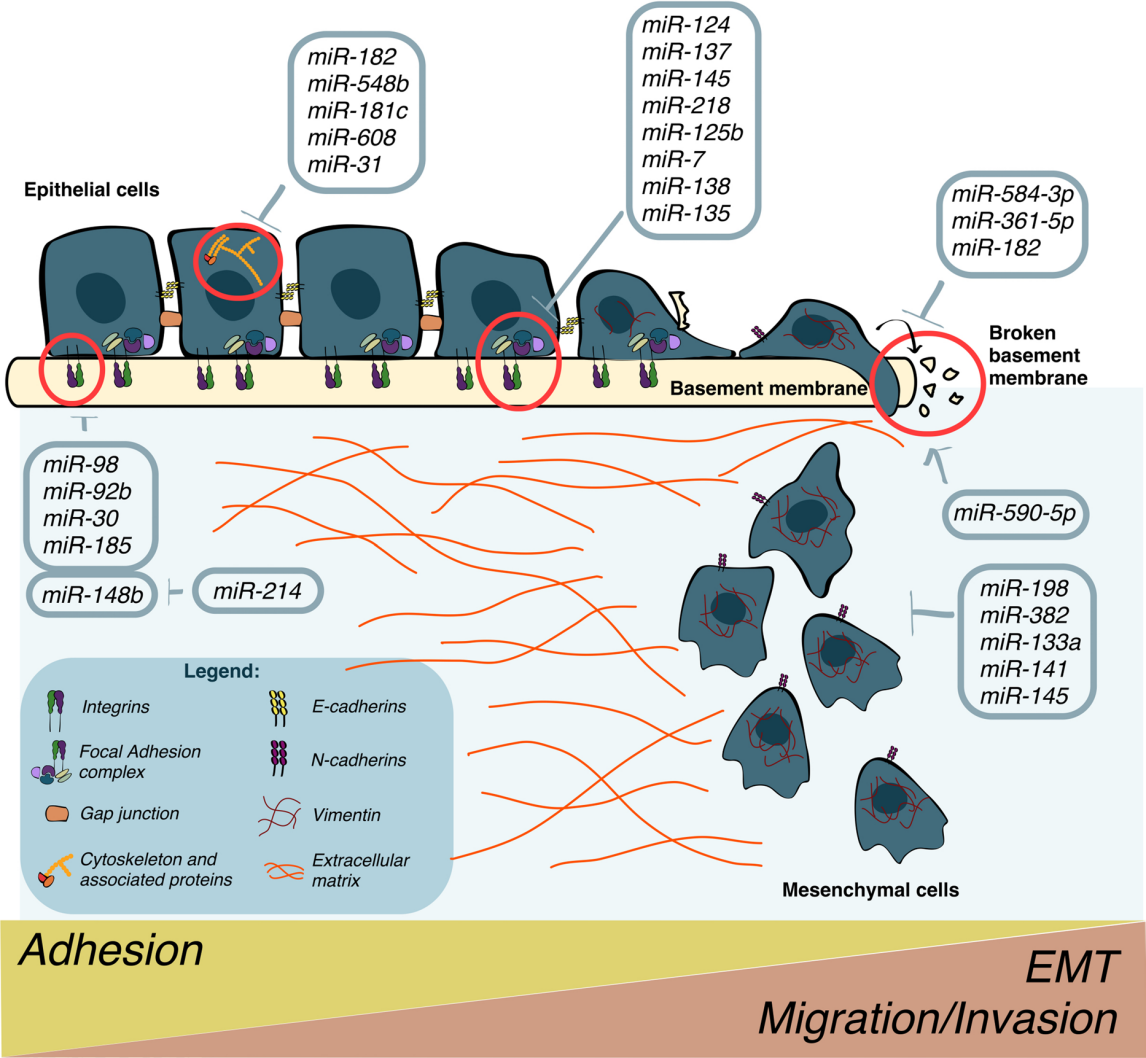
The adhesome is affected by miRNAs. The figure shows the miRNA-dependent post-transcriptional regulation of gene expression involved in adhesion and epithelial to mesenchymal transition (EMT)

Adhesome components are divided into distinct functional categories: adhesion receptors responsible for the ECM–cell signal transmission; actin regulating proteins controlling the communication between integrins and actin network remodeling; adaptor proteins that serve as hubs; kinases and phosphatases, mainly responsible for the phosphorylation or dephosphorylation of adhesome proteins, including GTPase activating proteins (GAPs) and guanine nucleotide exchange factors (GEFs) [73, 74]. All these proteins are regulated by miRNAs, as discussed below and summarized in Fig. 3.

#### Adhesion receptors

Among integrin heterodimers, the role of *α*5*β*1, *α*v*β*3 or *α*V*β*5 in the formation of fibronectin-dependent or independent adhesion complexes in cancer cells has been well explored [75, 76]. Various miRNAs target the single subunits of integrins: for example, miR-98 and miR-92b directly target ITG*β*3 and ITG*α*V in non-small cell lung cancer (NSCLC) and esophageal squamous cell carcinoma (ESCC), respectively [77, 78]. Sometimes, a single miRNA may target several heterodimers, as is the case of miR-30, capable of affecting *α*2*β*1, *α*5*β*1, *α*4*β*1 and *α*v*β*3 integrins, thus influencing bone metastasis formation [79]. In HCC, ITG*β*5 stabilizes *β*-catenin by enhancing its stability and here, miR-185 directly targets ITG*β*5. Relevantly, in these tumors, miR-185 expression is decreased compared to normal tissues while ITG*β*5 and *β*-catenin are increased [80]. Similarly, other pathways linked to integrins are involved in cancer progression, i.e., the miR-92b/ITG*α*6/Akt axis in ESCC [81] or the miR-214/miR-148b/ITG*a*5 axis in melanomas and breast cancers [82]. miR-214 is a pro-metastatic miRNA, while miR-148b exerts an anti-metastatic function in tumor progression even though they both act on adhesion receptors. As a matter of fact, miR-148b targets ITGA5 and ALCAM leading to dissemination inhibition. Conversely, miR-214 overexpression reverts the miR-148b-dependent phenotype by inducing miR-148b downregulation with the consequent derepression of ITGA5 and ALCAM [82]. These results suggest that adhesion receptors may be the final effectors of miR-on-miR pathways.

#### Adhesion complex regulators

When integrins bind to ECM proteins, the involvement of large protein complexes and signal transduction occur leading to the formation of various evolving complexes, such as primitive adhesions, focal complexes, FAs and fibrillar adhesions [83]. Several adaptor proteins, which regulate cell signaling and trafficking inside the cells, are able to localize in these complexes and to participate in the stabilization of signaling proteins [84]. Their loss of function leads to an upregulation of signal transduction, which can be detrimental for tumor progression [85]. The expression of some proteins which participate in the formation of these complexes is under miRNA control. Among them, talin, a protein which is controlled by miR-124 is capable of linking the cytoskeleton to the cell membrane by binding to actin filaments and to integrin cytoplasmic tails [86]. In PC, miR-124–talin interaction exerts an antitumoral effect by reducing cell adhesion, migration, and invasion *via* integrins and the FAK/Akt pathway and an inverse correlation between these two players was found in clinical specimens [87]. Paxillin, located at the interface between the plasma membrane and the actin cytoskeleton, is controlled by miR-137, miR-145, miR-218 and miR-125b [88, 89]. p140Cap, a multisite docking protein co-distributed with cortical actin and actin stress fibers and absent in FA [90], is regulated by several miRNAs [91].

Cortactin (CTTN), a protein which enables the formation of actin filaments at the leading edge of migrating cells, is controlled by miR-182 and its levels are inversely correlated with this small non-coding RNA, in NSCLC [92]. Actinins are proteins involved in filament crosslinking processes and the isoform alpha-actinin-4 (ACTN4) is inversely correlated with miR-548b in OSCC [93] and regulated in CRC by LIM domain kinase 1 (LIMK1) [94]. Actin remodeling plays an important role in the development of brain metastases. Tominaga et al. have shown that secretion of miR-181c *via* EVs promotes the destruction of the blood–brain barrier (BBB) through the downregulation of 3-phosphoinositide-dependent protein kinase-1 (PDKP1) and phosphorylated cofilin, causing the cofilin-induced modulation of actin dynamics [95]. Cofilin expression can also be altered by the small GTPase Rac2, a regulator of various proteins involved in actin remodeling and miR-608 has been found to be linked to Rac2 regulation in PC, where it is overexpressed [96]. Protein phosphorylation and dephosphorylation coordinate cellular communications and signaling by leading to conformational changes which, in turn, allow specific protein–protein interactions, therefore any alteration of phosphatase or kinases can be detrimental for cells and can lead to malignancy [97]. Among these enzymes, the Focal Adhesion Kinase (FAK), downstream of integrins, is tightly regulated by miR-7, miR-138, and miR-135 [98]. Finally, several GEFs and GAPs regulate Rho GTPases, thus impacting cytoskeleton modulation [99]. Interestingly, various miRNAs modulate GTPases such as miR-31 in glioma tissues [100] and miR-21 in CRC [101].

Overall, these data suggest a role for miRNAs in modulating tumor progression through the regulation of different proteins linked to adhesome formation and cytoskeletal modulation.

### Cellular protrusions and ECM degradation

Migration pathways are aberrantly regulated in cancer cells in which dissemination is favored by Epithelial to Mesenchymal Transition (EMT). Cells undergoing EMT lose their epithelial morphology to assume fibroblast-like structures and form protrusive and invasive structures that guide ECM remodeling, cell extravasation and organ colonization. The modulation of cell motility genes and miRNAs is a common trait of cancer dissemination [102]. Studies on HNSCC have revealed reduced levels of miR-198, able to target Daphnetin 1 (DAPH1), a protein that promotes directional migration by sequestering Arpin, a competitive inhibitor of the Actin-related proteins (Arp2/3) complex. Therefore, a reduction of miR-198 allows for an increase of actin filament branching and elongation, consequently enhancing migration and metastasis dissemination [103]. Similarly, miR-382 downregulation was observed by analyzing 200 melanoma samples and its depletion in melanoma cells revealed its anti-metastatic function. In fact, reduced levels of miR-382 in cells led to an increased expression of CTTN, Rac1 and ARPC2, all players of actin cytoskeleton remodeling. In particular, CTTN regulates lamellipodia and invadopodia formation and its depletion mimics the beneficial effects of miR-382 overexpression [104]. Another component of the Arp2/3 complex, ARPC5, is silenced by miR-133a and decreased in human patients with Head and neck sqaumous cell carcinoma (HNSCC) compared to controls [105]. miR-141 has also been identified as an ARPC5 regulator. Analysis of human xenograft prostate tumors in mice revealed a decrease in miR-141 related to higher amounts of the pro-metastatic Cdc42, Rac1 and ARPC5 [106]. Overall, studies investigating transcriptional and protein levels of Arp2/3 components in pancreatic, colorectal and breast carcinomas have shown contradictory results and in many cases an increase in gene expression has been reported which is directly related to tumor invasiveness. Other investigations however, revealed opposite results [107], suggesting that Arp2/3 upregulation occurs only in specific steps of tumor progression [108]. On the other hand, fascin overexpression is frequently accompanied by specific miRNA downregulation in cancer cells. For instance, miR-145, which is able to target fascin is significantly reduced in CRC, where metastases inversely correlates with miR-145 [109]. Malignant cells also need to degrade ECM in order to metastatize and this comes about by the release of metalloproteinases (MMPs) that are also regulated by miRNAs. For instance, MMP2 and MMP9 activity is enhanced by miR-590-5p overexpression in CRC xenografts, and its inhibition leads to decreased liver metastases. Moreover, miR-590-5p is upregulated in CRC patients and induced by the hypoxic TME [110]. By contrast, miR-584-3p inhibits gastric cancer (GC) progression by suppressing Yin Yang 1 (YY1), which binds to MMP14 promoter to improve its expression [111]. In breast cancer, miR-361-5p directly targets MMP1, thus reducing cancer cell invasion and is downregulated in tumor samples [33]. Additionally, miR-182 exerts an anti-metastatic role in NSCLC by targeting CTTN, thus affecting invadopodia formation [112]. Overall miRNAs play an essential role in the regulation of cellular protrusions and ECM degradation.

## The crosstalk between metabolism and adhesion in tumor progression

While the involvement of metabolism or adhesion components in cancer progression has been widely studied, their crosstalk still needs better investigation. Here, we discuss how alterations in the adhesion process may influence metabolic pathways and *viceversa*.

### Changes in adhesion can alter metabolic pathways in cancer

FAK interacts with integrins and growth factor receptors, thus affecting motility, growth and, ultimately, cancer progression. Growing evidence supports the link between FAK hyperactivation and aberrant metabolism in tumorigenesis which may promote glucose consumption, lipogenesis, and glutamine dependency [113]. In addition, its activation can promote aerobic glycolysis and tumorigenesis in PDACs by increasing the glycolytic genes enolase, PKM2, LDHA and reducing the OXPHOS. Conversely, FAK inhibition resensitizes cancer cells to growth factors, decreases cell viability and reduces tumor growth [114]. In GC, FAK, together with ITGB4, SOX2 and HIF1*α*, is part of a signaling pathway induced by the Extracellular Matrix Protein 1 (ECM1), which controls metastases and glucose metabolism [115]. In lung adenocarcinomas, FAK1 is negatively regulated by NeuroFibromin 1 (NF1), leading to metabolic rewiring. NF1 loss specifically causes FAK1 hyperactivation and accelerates murine *Kras*‐driven tumorigenesis. Tumors with NF1 mutations, addicted to glutamine, are susceptible to glutaminase and phosphoserine aminotransferase 1 (Psat1) inhibitors. This strategy could also be applied to other tumors with alterations in the NF1-FAK1 pathway [116]. All this evidence demonstrates the therapeutic potential of FAK inhibition. On the other hand, Demircioglu et al. showed that FAK depletion in CAFs enhances malignant cell glycolysis and tumor growth, thus indicating that FAK modulation in stroma cells may also affect cancer metabolism and progression [117].

The detachment of cancer cells from the ECM is also able to influence cell metabolism. In fact, Jeon et al. reported that matrix detachment of lung cancer cells induces a decrease in glucose uptake, activates LKB1 and AMPK, inhibits Ac-CoA carboxylases 1 and 2 which in turn, decreases NADPH consumption in fatty acid synthesis (FAS) while increasing NADPH generation through FAO fueling [118]. Similarly, also cell–cell adhesion, mediated by cadherins, is widely known to be critical for tissue homeostasis and maintenance of cell polarity. In particular, E-cadherin, which inhibits invasion, is lost during the EMT, in which a switch from E-Cadherin to N-Cadherin is observed. The presence of E-cadherin is typical of an epithelioid and well-differentiated phenotype and functional cell–cell junctions [119]. While EMT is responsible for cancer metabolic reprogramming towards an increase of glucose metabolism, the specific role of E-cadherin in cancer metabolism is not clear [120]. For instance, in breast cancer, E-cadherin has an unexpected regulatory ability in tumorigenicity and hypoxia responses: E-cadherin loss is associated with slower tumor growth and loss of hypoxia response genes, which lead to reduced glycolytic capacity. Moreover, high levels of E-Cadherin in basal breast cancers are linked to a poor clinical outcome [121].

The papers presented so far described how adhesion molecule alterations affect metabolism in cancer cells.

### Changes in metabolism can alter cell adhesion in cancer

Adhesion may not only alter metabolic pathways as described in the previous paragraph, but could itself be affected by tumor metabolism, which adds to the complexity of the cancer scenario. An example is represented by Ac-CoA, which alters adhesion genes in glioblastoma. Its changes affect the epigenetic modification H3K27ac, resulting in cell adhesion modulation through the activation of Ca2^+^–NFAT signaling, as demonstrated by Lee et al. Analysis of xenografts have confirmed the modulation of a panel of adhesion and migration-related genes in the absence of ATP citrate lyase (ACLY) [122]. We previously discussed how FAK may coordinate tumor progression by promoting glycolysis, but, in turn, glycolysis can modulate FAK *via* PEP which acts as a phospho-donor for histidine-58 in ESCC. Consequently, the PI3K-AKT signaling pathway is activated. Interestingly, ESCCs, but not esophageal adenocarcinoma cancer (EAC) cells, use the described pathway to induce growth factor-independent proliferation and, at the same time, to avoid growth factor signaling targeting therapeutics [123]. FAK may also be controlled by long-chain fatty acid CoA synthetase 4 (ACSL4), an enzyme involved in the conversion of fatty acids to fatty acid-Coenzyme A esters that may affect FAK protein stability. In GC, the expression of ACSL4 is downregulated in cancer tissues when compared to the adjacent mucosa and its decrease corresponds to FAK increase, while the levels of PTEN, vimentin, *β*-catenin remained unchanged [124]. Specific metabolic changes involving glucose, amino acid and lipid metabolism may alter E-cadherin expression, thus inducing EMT in cancer cells [125]. As a matter of fact, in laryngeal squamous cell carcinoma tissues, EMT markers are controlled by GLUT1, whose expression is positively correlated with vimentin and N-cadherin levels and negatively correlated with E-cadherin. These modulations could also have a prognostic value: in fact, high GLUT1, Vimentin and N-cadherin expression lead to a shorter survival rate in patients and those which, on the contrary, display high E-cadherin levels have a longer survival rate [126]. In NSCLC, EMT-associated genes, including E and N-cadherins, are regulated by alpha-enolase (ENO1), a key glycolytic enzyme, whose expression is increased in NSCLC tissues compared to normal ones, thus favoring glycolysis. Conversely, ENO1 downregulation increases E-cadherin expression [127]. Another pathway involved in the control of EMT is the TCA cycle. In nasopharyngeal carcinoma, TCA metabolites may cause IKK*α* recruitment to the promoter of EMT genes with the consequent decrease of E-cadherin and ZO-1 expression and an increase of vimentin, thus favoring EMT and metastases [128]. In PC, NAD, involved in glycolysis and TCA, controls EMT by influencing SIRT1, a NAD-dependent histone deacetylase capable of controlling epithelial morphology, E-cadherin transcription and mesenchymal marker levels, thus influencing cell adhesion [129]. In recent years, metformin, a common anti-diabetic drug, has shown its potential as a tumor protective drug. To dissect the anti-cancer mechanism, Banerjee et al. studied its involvement in EMT modulation, revealing an induction of MET and an upregulation of epithelial markers. In particular, metformin activates AMP-activated protein kinase (AMPK), leading to Snail and Slug suppression and E-cadherin upregulation. Interestingly, these results were also confirmed in the blood of diabetic patients undergoing metformin therapy, thus underlying the therapeutic relevance of a highly used metabolic drug useful in controlling tumor progression [130].

In conclusion, the papers presented so far show the existence of a crosstalk between metabolic pathways and the adhesion machinery in cancer. However, further in vivo investigations are necessary to better demonstrate these mutual regulations and their therapeutic value.

## The interplay between miRNAs, metabolism and adhesion/migration/invasion-mechanistic approach

**Fig. 4:**
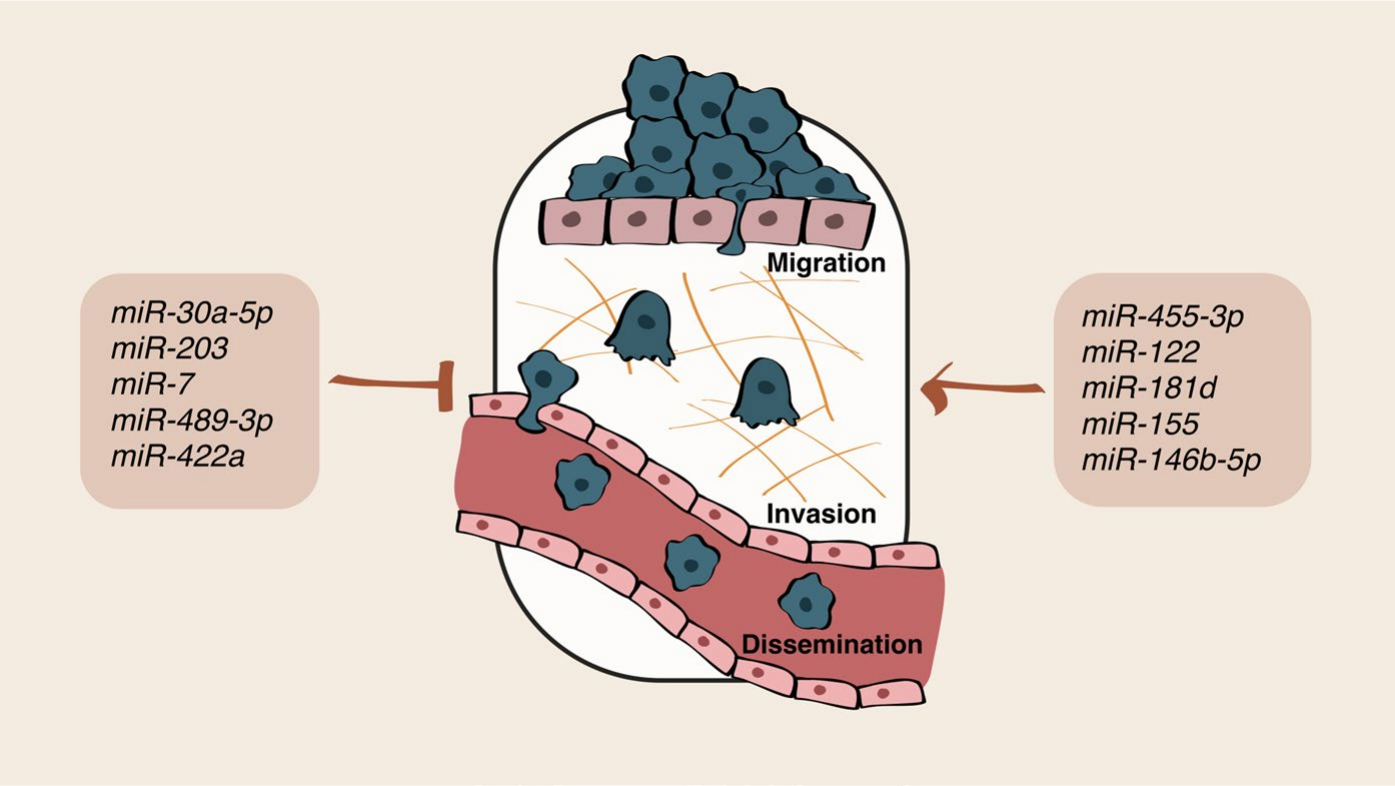
miRNA involved in metabolism and in adhesion/migration/invasion affecting tumor dissemination. This schematic drawing illustrates miRNAs relevant for metabolism and adhesion/invasion linked to tumor progression and dissemination

The alteration of energetic pathways are well-known hallmarks of cancer regulated by miRNAs as described previously. Here, we discuss miRNAs affecting players important both for metabolism and adhesion/invasion that impair tumor progression (Fig. 4).

miRNA modulation of glycolysis is able to influence dissemination in several ways. miR-455-3p, controlled by taurine upregulated gene 1 (TUG1), represses AMPKb2 expression and contributes to increased levels of Snail and HK2, thus leading to enhanced motility and invasion and glycolysis of hepatoma cells [131]. Interestingly, breast cancer cells may impair the utilization of nutrients by other cell types to favor themselves. As a matter of fact, tumor cells at the primary site may suppress glucose uptake in non-tumor cells of the metastatic niche through the secretion of vesicles with high miR-122 levels that target PK. Consequently, when miR-122 is inhibited, glucose uptake is restored in distant organs and the incidence of metastasis is reduced [132]. Also in breast cancer, miR-30a-5p acts through the inhibition of LDHA expression, leading to a decreased glucose uptake, lactate production, ATP generation, and ECAR as well as an increased OCR. Lung metastatization is strongly impaired following miR-30a-5p expression or LDHA knockdown in mouse tumors. Moreover, in breast cancer patients, miR-30a-5p negatively correlates with LDHA expression and increases FDG uptake [133].

In HNSCC, miR-203 blocks post-extravasation events during lung dissemination without affecting carcinoma differentiation in vivo. miR-203 controls metastasis through the regulation of 3 different players, including the metabolic gene NUAK1, a member of the AMPK catalytic subunit family involved in the maintenance of glycolysis [134]. miR-181d promotes aerobic glycolysis by protecting c-Myc from FBXL3 and CRY2-mediated degradation, which is responsible for CRC metastases. c-Myc, in turn, upregulates miR-181d and inhibits the expression of FBXL3 and CRY2, giving rise to a feed-forward loop [135]. To survive in a harsh environment characterized by hypoxia, starvation and reduced vascularization, PDAC cells increase their glycolysis and lactate production. Meanwhile these prohibitive conditions could activate autophagy. miR-7 represses autophagy and reduces the source of intracellular glucose to feed aerobic glycolysis by upregulating LKB1-AMPK-mTOR signaling, thus reducing proliferation and dissemination of cancer cells [136].

As previously said, miRNAs may act on TFs including SIX1, directly targeted by miR-489-3p, which, in turn, impairs glycolysis, decreases glucose uptake, lactate production, ATP generation, and ECAR, and increases OCR. This axis is relevant for melanoma dissemination in animal models as well as in patients who display an inverse correlation between miR-489-3p and SIX1, increased glucose uptake and metastases [137]. miR-155 activates STAT3, thus promoting HK2 transcription in breast cancer cells. The activation of this axis leads to higher glycolysis and a subsequent increase in the ability of cancer cells to disseminate [138].

The TCA cycle occurs in the mitochondria and oxidates the Ac-CoA derived from carbohydrates, fats and proteins, thus combining several metabolic ways. In GC, the production of Ac-CoA depends on the decarboxylation of pyruvate by the pyruvate dehydrogenase (PDH) whose levels may be restored after PDHK2 repression by miR-422a in GC. miR-422a overexpression in GC impairs malignancy and leads to a metabolic shift from aerobic glycolysis to oxidative phosphorylation. In addition, the miR-422a–PDHK2 axis promotes de novo lipogenesis and elevates the reactive oxygen species (ROS), arrests cell cycle in G1 phase and influences dissemination [139]. High miR-146b-5p levels promote cell growth, invasion and glycolysis. miR-146b-5p targets pyruvate dehydrogenase B (PDHB), whose overexpression leads to the termination of miR-146b-5p-mediated effects on growth, invasion and glycolysis [140].

Overall, miRNAs effect on both metabolism and adhesion of tumors or tumor-associated cells has a deep impact on multiple aspects of cancer biology, including cancer dissemination.

## Conclusions

Growing evidence supports the relationship between miRNAs and metabolism or metabolism and adhesion or miRNAs and adhesion. Here, we discussed the interconnections between the three different aspects, miRNAs/metabolism/adhesion, and how the different players may affect tumor progression based on the in vitro and in vivo data. The numerous tumor and stroma cell modifications and their connections occurring during cancer progression were highlighted. In particular, we presented data relative to miR-455-3p, miR-122, miR-30a-5p, miR-203, miR-181d, miR-7, miR-489-3p, miR-155, miR-422a and miR-146b-5p. Interestingly, the main control of these non-coding RNAs focuses on the glycolytic pathways, thus emphasizing the strong involvement of glucose metabolism in cancer. Nevertheless, further studies need to be performed to reinforce the connection between miRNAs, metabolism and adhesion/metastases in patients. The analyzed papers have demonstrated the relevance of miRNAs in this intricate network and these small non-coding RNAs consequently emerge as promising therapeutic candidates. In fact, miRNA expression and activity can be successfully modulated through miRNA mimics or inhibitors to replenish tumor suppressor miRNAs or inhibit oncomiRs, respectively. Moreover, considering the relevance of the discussed adhesion molecules and metabolic players, additional therapeutic interventions must be considered. Thus, from the content of this review new combinatorial therapies to reduce or eliminate cancer dissemination can be envisaged.

## Data Availability

Supplementary data to this article can be found available in the online version. The data underlying this article will be shared on reasonable request to the corresponding author.
